# Addressing current challenges in adult nursing: Describing a virtual Consensus development project methodology

**DOI:** 10.1002/nop2.1072

**Published:** 2021-09-25

**Authors:** Bethany Taylor, Jane South, Steve Robertson, Tony Ryan, Emily Wood, Rachel Louise King, Michaela Senek, Angela Tod, Jane Seymour

**Affiliations:** ^1^ Division of Nursing & Midwifery Department of Health Sciences University of Sheffield Sheffield UK; ^2^ School of Health & Community Studies Leeds Beckett University Leeds UK

**Keywords:** adult nursing, Consensus methodology, United kingdom

## Abstract

**Aim:**

This article describes the development and implementation of a virtual Consensus development project to address current challenges in adult nursing care in the UK.

**Design:**

This is a Consensus Development Project (CDP).

**Methods:**

The five stages of this CDP were: develop questions (informed by PPI representatives and a documentary review), generate evidence reviews, recruit and orient the lay panel, host Consensus seminars, and consult with panel members and stakeholders.

**Results:**

To the best of our knowledge, a CDP has not previously been conducted in a UK nursing context, and this is the first of its kind to be hosted virtually. This article contributes a detailed outline of the Consensus development methodology and constructive commentary to support future Consensus development projects. Learning points include reflections on the impact of hosting this event virtually, the relationship between the project coordinator and chair, and the composition of the lay panel.

## 1 INTRODUCTION/AIM

The aim of this paper is to describe a Consensus Development Project (CDP), which focusses on current challenges in adult nursing care in the UK. Consensus projects are aimed at generating discussion across public and academic networks in order to generate recommendations for practice and policy. These recommendations will further be used for engagement with the wider public, scientific community, nursing stakeholders and decision‐makers in policy and practice. As described in detail in the following section, Consensus approaches therefore provide an excellent method for those working in a range of international health care contexts (including nurses) to fully engage stakeholders in the development of appropriate and relevant services that will meet their needs. However, the practical aspects of organising, conducting, and reflecting on Consensus development work are often not well described. This paper outlines the implementation of this CDP; a methodology which, to the best of our knowledge, has not previously been conducted in a UK nursing context and is the first of its kind to be hosted virtually. The article describes the development and implementation of this CDP, outlining key stages and considering learning points. While the outcomes from this CDP are UK specific, insights about the process, particularly when undertaken virtually, will have wider international relevance for nurses who are considering using Consensus development approaches. The Consensus statement has been reported separately.

## 2 BACKGROUND

Consensus methods provide a means of informed decision making that is particularly valuable when evidence is lacking or inconsistent (Jones and Hunter, 1995). These methods draw on the views of experts, interested citizens, and other stakeholders, as well as existing evidence, to reach a Consensus (Hutchings & Raine, 2006). Consensus development methods highlight areas of debate and launch fruitful discussion to seek clarity and develop a Consensus on a particular issue (Kea & Chih‐An Sun, 2015). Although there is evidence of the use of Consensus methods in health care practice, relative to other sectors, their potential in this field has not yet been fully utilised (Kea & Chih‐An Sun, 2015).

This CDP was informed by a Canadian “Palliative Care Matters” initiative which hosted a Consensus development conference to decide the steps required to ensure that Canadians can access high‐quality palliative care services as part of their universal health care model (Covenant Health Palliative Institute, 2016; Fassbender, 2018). The CDP reported in this article was conceptualised according to the Consensus development conference model (Grundhal, 1995), utilising similar principles of engagement as the Canadian initiative (Covenant Health Palliative Institute, 2016; Fassbender, 2018). Whilst the concept of Consensus development was first posed in the 1950s, the idea that the process can be centred on a conference was introduced by the National Institute of Health in the USA in 1977 (Institute of Medicine (US) Committee to Improve the National Institutes of Health Consensus Development Program, 1990) and has since been used in other countries worldwide, each underpinned by the same principles but not necessarily following the same format.

The purpose of a Consensus development conference is to bridge the gap between the general public, policy‐makers, and “experts” (Grundhal, 1995). Civic involvement and the adoption of deliberative and inclusive approaches are increasingly recognised as essential and growing components of research, aiming to shape healthcare policy and service delivery (South et al., 2011; Street et al., 2014). Such involvement must be informed, effective, and meaningful to enable active and engaged citizens to contribute to decision making while also finding the experience stimulating and enjoyable (Abelson et al., 2003). This CDP contributes and supports this developing field by facilitating and prioritising meaningful deliberation between “interested citizens” and experts.

A suitable topic for a Consensus development conference is one that is topical, requires clarification but for which some evidence and expertise is available and lends itself to debate (Grundhal, 1995). Consistent with this definition, the topic chosen for this CDP was addressing current challenges in adult nursing care in the UK.

This CDP was intended to be hosted as a two‐day face‐to‐face conference; however, changes to this format were required in response to restrictions imposed during the Covid‐19 pandemic. The face‐to‐face conference event was cancelled and the programme was redesigned and hosted virtually. Rather than one event, the alternative design involved five virtual Consensus seminars hosted over a 2‐week period in October 2020. All interaction between the research team, the lay panel, and expert reviewers proceeded virtually.

Ethical approval was not required for this project. Within the UK context, work focused on obtaining views on research evidence and advising on its implementation does not always constitute research requiring formal ethics approval (though we recognise that this may vary in different international contexts). Nevertheless, this CDP did conform to the general ethics principles required by the university that led the work [University of Sheffield].

## 3 DESIGN AND METHODS

**FIGURE 1:**
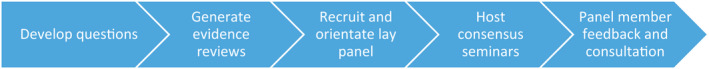
Stages of the Consensus development project

There were five stages to this CDP (see Figure 1.); developing questions, generating evidence reviews, recruiting and orienting the lay panel, hosting Consensus seminars, and consulting with panel members and stakeholders prior to publishing the Consensus statement. These will now each be described in turn.

## 4 DEVELOPING QUESTIONS

**FIGURE 2:**
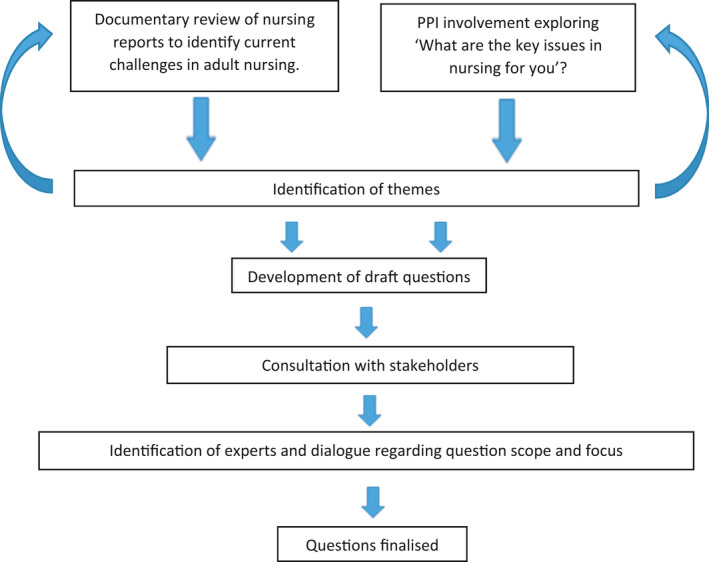
A flow diagram documenting the question formation process

It is essential to develop questions with unambiguous wording to optimise clarity and understanding (Fink et al., 1984) and to therefore enable maximum policy impact. The process by which questions were formed is shown in Figure 2. This involved engagement with key reports and documents, patient and public involvement (PPI) groups, stakeholders and identified experts. Through this synthesis and consultation, topics and themes were identified and five questions subsequently developed.

### 4.1 Documentary review of nursing reports

A documentary review aimed to identify key issues and themes across reports considering current challenges in adult nursing in the UK. Three grey literature databases were searched: Open Grey, NICE, and Social Care Online. Alongside this, the websites of carefully selected organisations were searched for relevant reports. A combination of the search terms “nursing”, “workforce”, and “care” was used. The search was completed in 2018 and restricted to documents published within the previous ten years. A broad approach was taken when reviewing documents with no restrictions placed on care setting, clinical intervention nor disease. The documents simply had to consider current issues in adult nursing in the UK. Following these searches and subsequent reading of documents found, selected references were also sought, read, and if relevant, incorporated into the review. Sixty‐two documents were selected for inclusion. Each document was read and key challenges were recorded. A thematic synthesis of such key challenges was developed and used to inform question topic ideas.

### 4.2 Consultation

Key challenges identified in existing reports were used as a starting point during discussions with stakeholders who were asked to discuss these, drawing on their own expertise and experiences. In doing so, identified challenges were embellished.

A number of patient, service user and advocacy groups were consulted over a 3‐month period in late 2018. Groups were representative of patients and carers living with a range of conditions and impairments (palliative and end of life care, mental ill health, learning disabilities, memory loss, and dementia). The research team attended pre‐arranged group meetings, shared the findings from the review of nursing reports, and facilitated feedback exercises and discussion in accordance with the communication needs of each group. An online PPI panel also contributed to this process by email.

The main questions asked during this consultation stage were:

- What are the evidence gaps in terms of providing safe and effective nursing care?
- What are the key issues associated with safe and effective nursing care?
- What are your experiences of nursing care and how could these have been improved?

The research team also shared the key themes with, and invited feedback from, the Royal College of Nursing Strategic Research Alliance advisory group and two nurse committees; a group of mesothelioma clinical nurse specialists working across the UK, and the Royal College of Nursing (RCN) Professional Nurses Committee.

Following the review of published nursing reports and consultation exercises, five questions relating to “current challenges in adult nursing” were proposed:

- What are the best ways of informing and engaging the public about nursing?
- What is the impact of “missed nursing care” or “care not done” on adults in health care?
- What are the essential elements required for safe and effective nursing care provision for adults?
- What are the inhibitors and facilitators to practice person and relationship centred nursing care?
- How can nursing Continuing Professional Development best influence quality of care for patients?

### 4.3 Identification of experts and dialogue about question scope and focus

A suitable expert for involvement in a Consensus development conference is a person with strong communication skills who is able to articulate their understanding clearly, be open to alternative suggestions and viewpoints and have good up to date knowledge of the topic area (Grundhal, 1995). Experts were therefore identified and invited to participate in this CDP because they had these desired qualities, were all well published and recognised for their contributions in the field of nursing research, and importantly, had valuable expertise in relation to one of the five question topic areas.

Consensus methodology is most effective when topics are carefully defined so that they can be explored in a timely and economical way (Fink et al., 1984). Mindful of this, invited experts engaged in helpful discussion with the research team to refine and set the scope and focus of each question, drawing on their extensive experience and knowledge of the relevant fields.

Each proposed question was cross‐referenced with the 62 reviewed documents to ensure a traceable audit trail of the process underpinning the question formation process. Although these documents did not necessarily contain information in response to the five proposed questions, they raised issues leading to the development of each of the questions.

## 5 GENERATING EVIDENCE REVIEWS

Each question was allocated to an expert (in two cases two experts who had worked closely with one another for a number of years) who conducted a synthesis of international evidence and generated a report in response to their question. Experts were encouraged to include their own points of view and reflections. A standardised structure for the report was shared with experts and the suggested word length was 3–4000 words. A shorter lay summary of each report and a glossary of terms used were generated.

## 6 RECRUITMENT AND ORIENTATION OF PANEL MEMBERS

Existing literature suggests that recruiting “interested citizens” helps to alleviate the risk of individual attitudes taking precedent over the common objective of the panel; to reach Consensus (Grundhal, 1995). Information about the CDP was shared via service user networks and direct contact with patient groups across the four UK nations. Interested participants contacted the research team in response to a national advert. They were requested to provide some background information to explain their interest in the CDP and their perspective on nursing.

To optimise representation, we aimed to recruit a panel of people with different perspectives and experiences and of mixed age, gender, occupation, ethnicity and with geographical spread across the four UK nations. Eleven panel members were recruited and participated in the CDP. Panel members were required to:

- Engage with preliminary activities
- Attend two remote orientation events
- Read the summaries of all five expert reviews
- Thoughtfully and critically engage with one of the five reviews
- Work with fellow panel members to construct a series of questions to pose to the expert reviewer(s)
- Co‐lead the questioning for their allocated review during one of the five Consensus seminars
- Attend the other four Consensus seminars to contribute to discussion
- Evaluate the knowledge shared and agree on recommendations
- Comment on the draft Consensus statement.

Payment in line with the current INVOLVE guidelines (INVOLVE, 2016) was available to panel members.

To prepare the panel in advance of the Consensus seminars, two orientation events were hosted virtually. It was essential to provide the panel with sufficient information and relevant evidence to inform their judgements and so that they did not solely rely on past experiences (Fink et al., 1984). These two events are described as follows:

### 6.1 Orientation one

The first orientation event was designed to introduce the lay panel to one another and the CDP process. Prior to orientation event one, two short films were recorded and shared with panel members. The first recorded a conversation with a small group of nurses contemplating their day to day nursing roles, satisfactions, challenges and key areas for future nursing research. The second film was recorded with RCN policy development managers and outlined why policy is important and how evidence can shape nursing policy. The two films informed discussion activities around what nursing is and what was in scope in the CDP.

### 6.2 Orientation two

Prior to the second orientation, lay summaries of the evidence reviews generated by experts were shared with panel members. This orientation event facilitated discussion of these reports and reflection on the roles and contributions of panel members to help establish team working. Following orientation, panel members were randomly allocated one of the five reviews and asked to co‐lead on this review. Co‐leading entailed generating questions to ask of experts and leading discussion in the Consensus seminar. Panel members were provided with the lengthier, original reports for the seminar that they were leading on and encouraged to contact the project coordinator or chair to discuss or clarify any issues or concerns prior to the Consensus seminars. Informal meetings were requested by some panel members.

## 7 HOSTING THE VIRTUAL Consensus SEMINARS

Five Consensus seminars were hosted virtually over a 2‐week period. Each seminar began with time for the panel to convene, decide on the order of questions and reflect on the content of the expert's report. This time provided an opportunity to identify the most significant issues. The two or three lay panel members who had been asked to co‐lead the seminar discussion shared their initial reflections and the panel agreed the most significant points/questions which would be raised. Following this, the expert(s) joined the meeting and presented key messages from their report in an oral presentation. Leading panel members initiated questioning and discussion, with other panel members contributing supplementary questions and comments. A debrief then provided an opportunity for the chair to summarise key points of uncertainty and Consensus and also invite final comments.

The following describes who was present during each seminar:

- All lay panel members
- Experts attended the Consensus seminar in which they were presenting but did not attend the other four seminars.
- The Chair [JS], facilitated the seminars, ensuring all voices were heard and that interaction between attendants was inclusive and constructive. The chair worked with the panel to identify key messages emerging from discussions. As recommended in key literature (Fink et al., 1984; Grundhal, 1995), the chair was chosen for their expertise and experience in facilitation and effectively involving members of the public in research. While the chair has an interest in and commitment to person‐centred health services and enabling the lay/patient voice, she is not an “expert” in nursing.
- The Project Coordinator [BT] provided introductions, helped support participation of panel members, kept each seminar to time, managed the chat function and supported the chair.
- An IT technician was available to solve any technical issues.
- Two members of the research team [AT & SR] alternated their attendance across the five seminars and mapped key concepts to ensure that the depth as well as breadth of the discussion was captured.

Maintaining accurate records of the seminar discussion was important to ensure transparency (Grundhal, 1995) and to support the development of the Consensus statement. The content of each seminar was recorded in four ways:

- Video recording, using the record option available on the video conferencing platform
- Online chat. This included a combination of comments and questions made by panel members
- Detailed and analytical notes generated by two members of the research team [AT & SR] who mapped the key concepts presented and discussed over the course of each seminar
- Notes generated by the Chair [JS]. These recorded key messages expressed by the panel, including principles, recommendations and practical solutions. These notes were based on the key points reported back to panel members and “sense‐checked” in the seminar debrief.

## 8 PANEL MEMBER FEEDBACK AND CONSULTATION

Drawing on the four recording methods used, the research team and the chair generated a synopsis of each seminar. These were 3–4 pages long and included four sections: why the topic is important; what evidence was presented to the panel; what issues the panel discussed; and what the key messages were. These key messages included issues where there was broad agreement, where there was concern or focus from the panel or where there were embryonic recommendations for what needed to be done in this area.

Panel members were invited by email to review and comment on the evidence summary for the seminar that they co‐led. Following amendments, all five evidence summaries were shared with all panel members who were then asked to identify which key messages they considered to be most important and to provide final comments. Once finalised, these summaries were shared with wider stakeholder groups, targeted for their knowledge and experience of the topics included in this CDP. Stakeholders were asked to consider: prioritisation, potential implications, policy relevance, and communication of message.

Prior to the virtual re‐design of the project, it was intended that stakeholders attend the conference event and contribute to discussion. Inviting stakeholders to respond to these questions enabled stakeholder involvement in the virtual CDP and was an important component of the process, recognising alternative viewpoints and increased the validity of the Consensus statement (Kea & Chih‐An Sun, 2015).

## 9 THE Consensus STATEMENT

This process has resulted in a Consensus statement that summarises potential policy, practice and research recommendations and can be used to lobby policy makers and inform decision making. Consistent with the principles of a Consensus development conference, the statement provides an authentic account of the issues raised and messages expressed by the lay panel (Grundhal, 1995). It has been designed to provide sufficient depth and breadth of information but in a short and accessible format, with links to supplementary documents to provide further detail about the CDP. While some findings included in the statement prompt clear action, others are more reflective and suggestive of a need for further evidence and consideration, but still provide a valuable contribution to addressing challenges in adult nursing in the UK.

## 10 DISCUSSION

This novel process was informed by best principles of Consensus development (Grundhal, 1995; Covenant Health Palliative Institute, 2016; Institute of Medicine (US) Committee to Improve the National Institutes of Health Consensus Development Program, 1990) in somewhat challenging circumstances; the switch to virtual methods. The remainder of this article focuses on a critical reflection of the process and methodology of the CDP which may help others in designing and implementing virtual Consensus events. These learning points consider the impact of hosting this event virtually, the relationship between the project coordinator and chair, and the composition of the lay panel.

## 11 IMPACT OF VIRTUAL COMMUNICATION

### 11.1 Accessibility

The use of virtual communication enabled panel members and experts, two of whom were international, to attend with no travel requirements or risk of exposure to Covid‐19. Nevertheless, the shift to virtual methods meant that one panel member could no longer participate due to hearing difficulties and associated anxiety about contributing via a virtual platform. Potential use of technology that enabled subtitles may have improved the accessibility of the CDP for this person and would be a valuable amendment to a future CDP.

One of the primary concerns throughout the project was internet/broadband connection issues causing potential disruption to the Consensus seminars. Fortunately, no major connection problems arose and the few minor issues that did were very quickly resolved. Advice and recommendations for conducting online events (National Co‐ordinating Centre for Public Engagement, 2020) were found to be exceptionally helpful. Strategies to reduce the risk of disruption due to connection problems included allowing extra time for panel members to join the meeting prior to the event starting, having an IT technician “on call” for support and sharing detailed and clear instructions with attendants. Optional “rehearsal” meetings were also available to panel members to ensure they were able to access the virtual platform confidently.

### 11.2 Team working

It was important that panel members shared a common quest for Consensus and did not try to canvas support for their own points of view. Enabling panel members to work collectively in this way was perhaps more difficult in a virtual setting than it would have been in a face‐to‐face environment. This was principally because the virtual setting did not lend itself to the more informal face‐to‐face discussion that one may share if attending an event in person. The orientation events were originally planned to be hosted over 2 days, 4 weeks apart, in a conference hotel venue. Time during breaks and over lunch would have enabled panel members to interact informally on a one‐to‐one basis. The circumstances did not allow for this and much shorter periods of time were allocated for the virtual orientation events to reduce the risk of screen fatigue. While it is not possible to know the exact impact of the virtual environment on the ways in which the panel worked together, establishing a sense of togetherness amongst the team was perceived to be more difficult. Nevertheless, the panel did orientate quickly to each topic and engaged well with the process. The orientation events were a valuable means of introducing panel members to one another, the CDP process and the technology used. On reflection, perhaps an additional third orientation event and/or more icebreaker activities could have been incorporated. These activities could have even been completed prior to or in between orientation events using a blog or other online space.

When sharing the evidence summaries, it was important to achieve the delicate balance between providing sufficient detail and not overwhelming panel members with information. The decision to allocate panel members one of the five reviews assisted in achieving this balance. This meant that panel members were asked to read all five review summaries but only one review in full. This allocation also encouraged interaction between panel members away from the larger group meetings. The two or three panel members allocated to each review communicated with one another, either by email, phone or video call to discuss their allocated review and potential questions. Sharing the responsibility in this way encouraged panel members to contribute and alleviated the potential for more confident characters to dominate discussion and the design of questions. There was evidence of in‐depth engagement with the evidence from all panel members.

### 11.3 Ground rules

Orientation events required skilled facilitation, in part due to the enthusiasm of some panel members for the subject matter and process. The absence of body language cues typically used to divert attention from one panel member to another, or to encourage further comment, meant that chairing the events was particularly, and perhaps at times unforeseeably, challenging. In this way, the orientation events proved to be preparation for the project coordinator and chair as much as they were for panel members. These events enabled the chair and project coordinator to get to know the panel and establish strategies and confidence to enact ground rules. These ground rules included not speaking over other panel members, raising a (virtual) hand when wishing to speak and using the mute button when others were speaking. On reflection, the virtual orientation events were essential to ensuring all panel members were involved in co‐producing a set of ground rules that could be committed to.

### 11.4 The pressure of time in a virtual environment

While the face‐to‐face events would have required panel members to travel and commit two full days to their involvement, the switch to virtual engagement requested time over a 2‐week period which may have placed greater expectations on the panel and caused more inconvenience than the original 2‐day conference. The length of virtual events was restricted to 2 h, mindful that panel members may experience screen fatigue. Consequently, the virtual format was potentially more convenient for some panel members who had busy lives with family and/or work commitments. In addition, the time between Consensus seminars did enable greater preparation and reflection than would have been possible during the face‐to‐face event. Throughout each seminar, all attendees were observably energetic and bright without the usual signs of fatigue as expected at the end of a long day at a conference.

Nevertheless, a significant challenge of the virtual events was the lack of time to deliberate all issues and pinpoint a clear Consensus. Time is a recognised limitation to reaching deliberation during face‐to‐face events (Smith & Wales, 2000), so it is understandable that the virtual environment can pose an additional challenge. In order to minimise the impact of this, evidence summaries were shared with panel members for comment following the seminars; however, this did require additional researcher time and resources. As well as this, the chair ended each session with a summary of issues that had been debated, emerging themes and invited comment on whether the summary reflected discussion. While capturing the essence of the seminar was a difficult and skilful task for the chair, this “testing” of emerging Consensus helped in developing later evidence summaries and was considered an essential element of the virtual Consensus seminars. There were clear key messages and some of these were cross‐cutting.

## 12 RELATIONSHIP BETWEEN CHAIR AND PROJECT COORDINATOR

It was clear throughout that the principal role of the chair was to facilitate and empower the panel to reach a Consensus in a fair and inclusive way, and to do so with no agenda regarding the outcome of the CDP. The close working relationship between the project coordinator and the chair proved valuable in achieving this and to a smooth and seamless CDP. For example, panel members sometimes contacted the project coordinator about issues and concerns, and it was important that the chair was aware of these. Open and constructive communication between the chair and the project coordinator was essential to ensure that panel members were best equipped and supported to contribute to this process. During the seminars, both the project coordinator and chair were committed to ensuring that panel members were prepared, confident and at ease. This was critical to building trust amongst panel members and contributed to the inclusive and open atmosphere required to facilitate genuine deliberation and consideration of important topics relevant to adult nursing care. For the purpose of this virtual CDP, the role of the chair needed to be less of an independent arbiter and more a facilitative bridging role, working closely with the project team to ensure inclusive deliberation. Feedback from panel members regarding the support they received was positive.

## 13 COMPOSITION OF THE LAY PANEL

The high level of panel member involvement optimises the reach and relevance of the Consensus statement and recommendations to patients and communities. We do however recognise that limitations to the diversity of the panel may have shaped these outcomes. While there is little evidence concerning the impact of lay panel characteristics on the outcomes of Consensus development methods (Hutchings & Raine, 2006), efforts should be made to optimise the diversity of the panel. The research team aimed to recruit a diverse panel of mixed gender, age, ethnicity and location, for instance, living across the four UK nations in rural and urban environments. Unfortunately, all panel members were white British and the panel only included representation from three of the four UK nations, with no representation from Northern Ireland. It can however be said that overall, the lived experience of panel members, some of whom had recent experiences as patients or carers, and one who had a learning disability, brought a richness to discussions. It was important for the chair to carefully ensure that these experiences were shared to illustrate and open up discussion rather than generalise based on a single event. This was managed well and skilfully by the chair but could have been a potential issue. In hindsight, use of social media to advertise panel recruitment may have increased the diversity represented on the panel.

## 14 CONCLUSION

This article has outlined the implementation of a Consensus Development Project (CDP) to address current challenges in adult nursing and provided a reflective account of key learning points. In doing so, this work makes a valuable contribution to the knowledge base in relation to CDP implementation methods. It is our understanding that this is the first CDP to be hosted using virtual methods and the first conducted in the context of UK nursing. Hosting this CDP virtually was not without its challenges, most significantly establishing a sense of togetherness amongst panel members, chairing seminars without the use of non‐verbal cues typically relied upon in face‐to‐face facilitation, and the short amount of time to pinpoint key areas of Consensus during seminars. Preparation and skilful facilitation to overcome issues such as these was fundamental to the success of this CDP. Nevertheless, the virtual CDP allowed attendants to take part from the comfort and safety of their own homes, increasing the diversity of those that could participate. Engagement throughout was inclusive, enthusiastic and productive. This CDP contributes to developments in research by demonstrating the richness of contributions made by citizens. The resulting Consensus statement and recommendations will be widely disseminated and used to inform nursing policy in the UK.

## CONFLICT OF INTEREST

The authors have no conflicting interests to declare.

## AUTHOR CONTRIBUTIONS

Bethany Taylor drafted the paper. Bethany Taylor and Jane Seymour conceptualised and designed the project. Bethany Taylor, Steve Robertson, Tony Ryan, Angela Tod, and Jane South contributed to the preparation and delivery of Consensus seminars. All authors contributed to the development of the protocol and the design and dissemination of the Consensus statement. All authors read and approved the final manuscript.

## Data Availability

Data sharing not applicable to this article as no datasets were generated or analysed during the current study.
